# Dietary milk fat globule membrane supplementation combined with regular exercise improves skeletal muscle strength in healthy adults: a randomized double-blind, placebo-controlled, crossover trial

**DOI:** 10.1186/s12937-015-0073-5

**Published:** 2015-08-25

**Authors:** Satoko Soga, Noriyasu Ota, Akira Shimotoyodome

**Affiliations:** Biological Science Laboratories, Kao Corporation, 2606 Akabane, Ichikai-machi, Haga-gun, Tochigi 321-3497 Japan

## Abstract

**Background:**

Our previous studies demonstrated that dietary supplementation with milk fat globule membrane (MFGM) combined with habitual exercise improved muscle strength by stimulating neuromuscular development in mice. This study aimed to demonstrate the beneficial effects of dietary MFGM supplementation plus regular exercise on muscle strength and neuromuscular function in healthy humans.

**Methods:**

The study was designed as a randomized, double-blind, placebo-controlled, crossover trial. Fourteen Japanese adults aged 31–48 years took daily MFGM (1 g) or placebo tablets during the 4-week study period and attended a training program twice a week. Physical function tests and surface electromyography (EMG) were conducted at baseline and at the end of the study period.

**Results:**

The MFGM group had significantly greater leg extension strength than the placebo group after the 4-week study period. Surface EMG showed that the MFGM group had a significantly higher root mean square amplitude than the placebo group, which indicated that the MFGM group had higher motor unit activity.

**Conclusions:**

Dietary MFGM supplementation combined with regular exercise improves skeletal muscle strength, which may be due to increased motor unit recruitment in healthy Japanese middle-aged adults.

## Background

Muscle strength is an excellent indicator of general health. Optimal muscle function is important with respect to rehabilitation and quality of life in many musculoskeletal diseases [1–4]. However, starting at the age of 25 years, muscle strength in healthy men decreases in a linear fashion, losing 54–89 % of its capacity by the age of 75 years [5]. Therefore, the decline of skeletal muscle strength in younger aged adults should be prevented to maintain a higher quality of life in their older age.

Resistance training is well known to increase muscle mass and strength [6]. However, high-intensity and long-term resistance training is necessary to achieve satisfactory improvement of muscle mass and strength. Accordingly, more efficient strategies that boost the exercise-induced improvement of skeletal muscles are required to maintain general health for non-athletes. Nutritional supplementation may facilitate more efficient muscle improvement for people lacking exercise or when combined with low-intensity and low-frequency exercise. Recent studies demonstrated that dietary supplementation with amino acids [7] or tea catechins [8] significantly improved muscle mass and strength in the elderly when combined with regular, low intensity exercise.

Our recent studies in mice have demonstrated that dietary supplementation with milk fat globule membrane (MFGM), when combined with habitual exercise, significantly improved muscle mass and strength [9], as well as swimming endurance capacity [10]. MFGM is the structural membrane covering a triglyceride globule that is dispersed as emulsified bodies in milk [11]. Our previous study revealed that the beneficial effects of dietary MFGM on skeletal muscles was associated with stimulation of neuromuscular junction (NMJ) development [9], which is a critical structure of a motor unit (a single motor neuron and all of the muscle fibers that it innervates). MFGM supplementation in the diet combined with voluntary exercise (wheel-running) significantly increased muscle strength and expression of NMJ-related molecules in adult mice [9]; however, whether nutritional supplementation with MFGM can facilitate neuromuscular improvement by regular exercise in humans has yet to be explored.

The present study aimed to investigate whether dietary MFGM combined with regular exercise can increase skeletal muscle strength and neuromuscular function in healthy middle-aged adults.

## Methods

### Subjects

Fourteen male subjects (aged 31–48 years) were enrolled in the present study (Table 1). Subjects were excluded if they had uncontrolled hypertension, coronary heart disease, or if they engaged in resistance training in their daily lives. They were instructed not to change their daily exercise habits or diets during the study period. Signed informed consent from each subject was obtained after fully informing them about the details and methods of this study. The study was performed under the supervision of an occupational health physician, in accordance with the regulations of the Kao Corporation Ethics Committee for Internal Clinical Studies and in conformity with the Declaration of Helsinki.

**Table 1:** Characteristics of study subjects

	Value
Age, year	39.9 ± 1.3
Body weight, kg	69.1 ± 2.7
Body fat ratio, %	18.7 ± 1.5
Whole BMM, kg	52.8 ± 1.3
Leg muscle mass, kg	9.7 ± 0.4
Thigh circumference, cm	46.4 ± 0.9

BMM, body muscle mass

Body weight, body fat ratio, whole body and leg (average of left and right) muscle mass were measured using a bioimpedance body fat analyzer (BC-621, Tanita, Co., Tokyo, Japan). Thigh circumference (average of left and right) was measured 15 cm proximal to the superior pole of the patella

Values are mean ± SEM (*n* = 14)

### Randomization and study protocol

The study was designed as a randomized, double-blind, placebo-controlled, crossover trial. Randomization was performed after baseline assessment. The randomization procedure was conducted by a person who was not involved in the study, and the subjects and test staff remained unaware of the assignments throughout its duration. The subjects were randomly divided into 2 groups. During the first 4-week intervention period, one group (*n* = 7) received placebo tablets and the other (*n* = 7) received MFGM tablets. Subjects took test tablets each day during the 4-week period (period 1) and underwent exercise training twice a week. A 4-week washout period was followed by a second 4-week intervention period (period 2) in which the groups were reversed. Physical function tests were performed at the beginning and the end of the 4-week intervention. We assessed the safety of the test tablets by evaluating the adverse events, discontinuation rate, and vital signs, as well as by conducting additional laboratory safety tests.

### Milk fat globule membrane consumption

Each subject ingested a MFGM tablet (1 g) or a placebo tablet (1 g whole milk powder) daily for 4 weeks. The daily dose of MFGM (1 g; equivalent to 600 mL of whole milk) was chosen based on what is known about its nutritional safety and efficacy, after converting the minimal effective dose in mice [10] to a human equivalent while considering the relative body surface areas. On the exercise training enforcement days, the subjects were instructed to take the tablet within 1 h before training. On the other days, the subjects were instructed to consume the tablet at the time of their choice during their daily routines.

The MFGM was prepared from buttermilk by filtering and centrifugation. The MFGM and whole milk powder compositions were analyzed at Japan Food Research Laboratories (Tokyo, Japan). The composition of the MFGM and whole milk powder are shown in Table 2.

**Table 2:** Composition of MFGM and whole milk powder

	MFGM, %	Whole milk powder, %
Protein	46.9	26.3
Fat	35.7	25.2
Carbohydrate	10.6	39.5
Phospholipids	16.6	0.286
Phosphatidylcholine	4.71	0.067
Phosphatidylethanolamine	5.20	0.063
Phosphatidylinositol	1.32	0.037
Phosphatidylserine	1.74	0.033
Sphingomyelin	3.62	0.057
Ash	3.8	5.7
Moisture	3.0	3.3

*MFGM* Milk fat globule membrane

### Exercise training

Training was conducted twice weekly on nonconsecutive days for 4 weeks using StrengthErgo 240 stationary cycling exercise machines (Mitsubishi Electric Corporation, Tokyo, Japan). The subjects completed 3 sets of 15 % maximal voluntary contraction (MVC) cycle exercises for 60 s and 7 sets of 20 % MVC cycle exercises for 40 s at 50 rpm.

### Physical function test

Physical function testing consisted of anthropometric measurements, blood analysis, and leg extension strength tests. Tests were conducted before and after the 4-week intervention period.

#### Anthropometric measurements

Body weight, body fat ratio, and muscle mass were measured using a bioimpedance body fat analyzer (BC-621, Tanita, Co., Tokyo, Japan). Thigh circumference was measured 15 cm proximal to the superior pole of the patella.

#### Blood sampling and analysis

Blood was collected from an intermediate vein of the forearm after an overnight fast for the following serum tests: aspartate aminotransferase (AST), alanine aminotransferase (ALT), glucose, triglyceride, and total cholesterol. The serum samples were analyzed at SRL Inc. (Tokyo, Japan). The numbers of white blood cells and red blood cells (RBCs), as well as the hemoglobin concentration, were measured with an automatic hemocytometer (Celltac MEK-5258, Nihon Kohden, Tokyo, Japan).

#### Leg extension strength

The isokinetic extension strength of each leg was the primary outcome and was measured using the StrengthErgo 240 (Mitsubishi Electric Co., Tokyo, Japan) [12, 13]. Each subject pedaled the apparatus with maximum effort 5 times. The peak muscle strength of the right and left legs was calculated as the maximal isokinetic leg extension strength.

#### Surface electromyography

Motor unit activity during the exertion of muscle force was evaluated using surface electromyography (EMG) [14, 15]. The surface EMG signals of the intermediate portion of the right vastus medialis were detected during leg extension strength measurement with the Ambu® Blue Sensor M (Ambu Ltd., Ballerup, Denmark). After preparing the skin with an alcohol swab to reduce impedance, an electrode was fixed on the skin surface so that it ran along the underlying muscle fibers. The EMG signal was measured using a Bio-monitor ME6000 (Mega Electronics Ltd., Kuopio, Finland). After the analog-to-digital conversion, the root mean square (RMS) amplitude, an indicator of motor unit activity [16, 17], was calculated using the MegaWin 3.0 software (Mega Electronics Ltd., Kuopio, Finland).

### Sample size and statistical analysis

The sample size of this crossover study was based on our previous study investigating leg extension strength after exercise intervention, where the goal was to detect a 5 % change [18]. As we used two-sided tests to achieve type I error rates of less than 5 %, we determined that 14 subjects would be sufficient to provide a statistical power of 80 % in these types of studies.

All variables are presented as mean ± SEM. The differences in study endpoints or the changes from baseline between interventions were analyzed by using a linear mixed model. We included the fixed effects of group, period, period baseline, and the interaction of group and period. We also included subject as a random effect. When the model showed a significant intervention effect, pairwise comparison between interventions was performed with Bonferroni analysis, and changes were considered significant at *P* < 0.05. For statistical analysis, SPSS for Windows, release 23.0 (SPSS, Chicago, IL) was used.

## Results

### Effects on anthropometric values and blood variables

All subjects completed the intervention protocol, and there were no adverse side effects from the ingestion of the test tablets. No overall changes in body weight, body fat ratio, whole body and leg muscle mass, or thigh circumference were observed during the intervention period (Table 3).

**Table 3:** Changes in anthropometric variables before and after the intervention

	Baseline		4 weeks	
	Placebo	MFGM	Placebo	MFGM
Body weight, kg	69.0 ± 2.7	69.5 ± 2.8	69.0 ± 2.7	69.5 ± 2.7
Body fat ratio, %	18.6 ± 1.6	17.7 ± 1.5	18.4 ± 1.6	18.3 ± 1.6
Whole BMM, kg	52.8 ± 1.2	53.8 ± 1.4	52.9 ± 1.2	53.3 ± 1.2
Leg muscle mass, kg	9.7 ± 0.36	9.9 ± 0.38	10.0 ± 0.34	10.0 ± 0.36
Thigh circumference, cm	45.8 ± 0.9	46.2 ± 0.9	45.8 ± 0.8	45.9 ± 0.9

*MFGM* Milk fat globule membrane, *BMM* Body muscle mass

Body weight, body fat ratio, whole body and leg (average of left and right) muscle mass were measured using a bioimpedance body fat analyzer (BC-621, Tanita, Co., Tokyo, Japan). Thigh circumference (average of left and right) was measured 15 cm proximal to the superior pole of the patella

Values are mean ± SEM (*n* = 14). There were no significant differences between the groups and the changes within each group from baseline

There were no clinically significant changes in fasting serum AST, ALT, glucose, triglyceride, or total cholesterol levels during the 4-week intervention (Table 4). RBCs and Hemoglobin levels after 4 weeks were significantly higher in the MFGM group compared with the placebo group, although the mean values did not deviate from the Japanese standard values (Table 3).

**Table 4:** Values of serum and blood components before and after the intervention

		Placebo	MFGM	*P*-value
Serum components				
AST, IU/L	Baseline	17.7 ± 1.5	17.1 ± 0.6	
	4 weeks	17.6 ± 0.8	16.6 ± 0.6	*P* = 0.354
ALT, IU/L	Baseline	14.3 ± 1.8	12.9 ± 1.3	
	4 weeks	12.4 ± 1.1	11.9 ± 1.0	*P* = 0.880
Glucose, mg/dL	Baseline	96.6 ± 2.1	95.5 ± 3.1	
	4 weeks	94.3 ± 2.0	97.7 ± 2.5	*P* = 0.092
Triglyceride, mg/dL	Baseline	99.4 ± 9.8	95.3 ± 10.5	
	4 weeks	92.5 ± 11.1	95.8 ± 9.2	*P* = 0.397
Total cholesterol, mg/dL	Baseline	187.9 ± 7.6	179.9 ± 7.7	
	4 weeks	177.7 ± 6.6	177.3 ± 7.4	*P* = 0.387
Blood components				
WBCs, 10^2^/μL	Baseline	49.6 ± 4.1	46.7 ± 3.5	
	4 weeks	41.9 ± 3.2	46.4 ± 2.9	*P* = 0.164
RBCs, 10^4^/μL	Baseline	503.1 ± 8.3	508.4 ± 8.9	
	4 weeks	494.0 ± 11.6	520.9 ± 11.1	*P* = 0.013
Hemoglobin, mg/dL	Baseline	15.1 ± 0.2	15.3 ± 0.2	
	4 weeks	14.8 ± 0.2	16.0 ± 0.3	*P* < 0.001

*MFGM* Milk fat globule membrane, *AST* Aspartate aminotransferase, *ALT* Alanine aminotransferase, *WBCs* White blood cells, *RBCs* Red blood cells

Blood was collected from an intermediate vein of the forearm after an overnight fast. The serum samples were analyzed at SRL Inc. (Tokyo, Japan). The numbers of WBCs and RBCs, as well as the hemoglobin concentrations were measured with an automatic hemocytometer (Celltac MEK-5258, Nihon Kohden, Tokyo, Japan)

Values are mean ± SEM of 14 subjects. Differences in study endpoints between interventions were analyzed by using a linear mixed model, with group, period, period baseline, and the interaction of group and period as fixed effects; and subject as a random effect. When the model showed a significant intervention effect, pairwise comparison between interventions was performed via Bonferroni analysis. Changes were considered significant if *P* < 0.05

### Effect on muscle strength and electromyography parameters

After the intervention, a significant difference was detected for leg extension strength (Table 5). The strength in the MFGM group was significantly higher than in the placebo group at 4 weeks. Moreover, the percent change from the baseline was significantly higher in the MFGM group than in the placebo group. Although the baseline of period 2 was higher than that of period 1 in this crossover study, this apparent carryover effect after the washout period was not significant.

**Table 5:** Values of leg extension strength before and after the intervention

		Placebo	MFGM	*P*-value
Leg extension strength, Nm	Baseline	177.6 ± 7.3	175.8 ± 8.0	
	4 weeks	175.7 ± 7.3	182.2 ± 7.0	*P* = 0.001
Change from baseline to 4 weeks, %				
	4 weeks	−0.95 ± 1.2	4.23 ± 1.3	*P* = 0.001

*MFGM* Milk fat globule membrane

The leg extension strength was measured using StrengthErgo 240 (Mitsubishi Electric Co., Tokyo, Japan)

Values are means ± SEM of 14 subjects. Differences in study endpoints between interventions were analyzed by using a linear mixed model, with group, period, period baseline, and the interaction of group and period as fixed effects; and subject as a random effect. When the model showed a significant intervention effect, pairwise comparison between interventions was performed via Bonferroni analysis. Changes were considered significant if *P* < 0.05

The percent change from baseline RMS, an indicator of motor unit activity during the leg extension strength measurement, was significantly higher in the MFGM group than in the placebo group (Table 6).

**Table 6:** Electromyogram results before and after the intervention

		Placebo	MFGM	*P*-value
RMS, mV	Baseline	0.319 ± 0.035	0.278 ± 0.032	
	4 weeks	0.293 ± 0.044	0.308 ± 0.034	*P* = 0.142
Change from baseline to 4 weeks, %				
	4 weeks	−8.92 ± 7.28	12.52 ± 5.80	*P* = 0.028

*MFGM* Milk fat globule membrane, *RMS* Root mean square

The surface EMG signals of the right vastus medialis were detected during leg extension strength measurement with the Ambu® Blue Sensor M (Ambu Ltd., Ballerup, Denmark). The EMG signal was measured using a Bio-monitor ME6000 (Mega Electronics Ltd., Kuopio, Finland). After the A/D conversion, the root mean square (RMS) amplitude was calculated using MegaWin 3.0 software (Mega Electronics Ltd., Kuopio, Finland)

Values are means ± SEM of 12 subjects. Differences in study endpoints between interventions were analyzed by using a linear mixed model, with group, period, period baseline, and the interaction of group and period as fixed effects; and subject as a random effect. When the model showed a significant intervention effect, pairwise comparison between interventions was performed via Bonferroni analysis. Changes were considered significant if *P* < 0.05

## Discussion

This study had two major findings. The first was that daily intake of 1 g MFGM combined with regular, twice weekly exercise improved skeletal muscle strength (leg extension) in middle-aged adults, despite a lack of change in muscle mass. The second was that dietary MFGM supplementation plus regular exercise also increased the RMS of surface EMG, indicating that dietary MFGM increased motor unit activity during muscle contraction.

Surface EMG comprises of the sum of the electrical contributions made by the active motor units, and its amplitude is related to the net motor unit activity (i.e., the recruitment and the discharge rates of the active motor units) [16, 17]. Improved neurological adaptation in skeletal muscles has been recognized to provide a larger proportion of the initial strength increment during isotonic strength training compared to muscle hypertrophy [19]. This muscle reinforcement is accompanied by increased RMS that indicates increased motor unit recruitment during neurological adaptation. Because the leg muscle mass did not change after MFGM ingestion in the present study, increased muscle strength by dietary MFGM supplementation appears to be due to the increase in motor unit activity such as recruitment.

Pathway analysis after transcriptomic measurement in our previous study [9] revealed that dietary MFGM combined with regular exercise improved muscle strength in adult mice primarily by stimulating the pathway involving “nervous system development” in the skeletal muscle. This pathway includes functional annotations such as formation of synapses, growth of neurites, or development of NMJ. Dietary MFGM combined with exercise increased skeletal muscle expression of docking protein-7 (Dok-7) and muscle-specific receptor tyrosine kinase in mice [9], both of which play a critical role in NMJ formation [20, 21]. Defects in NMJ function causes muscle weakness in neuromuscular disorders, and Dok-7 gene therapy improves NMJ formation and rescues the motor activity [22]. The results in the present study are consistent with the previous findings and indicate that dietary MFGM plus exercise increases motor unit recruitment and enhances muscle strength, probably owing to neuromuscular mechanisms.

In this study, the semi-weekly exercise alone did not increase muscle strength in the placebo group. This may be due to the moderate intensity of the training, which challenged only 15–20 % of their leg extension strength. Nevertheless, nutritional supplementation with MFGM significantly boosted muscle strength in middle-aged adults completing this moderate intensity semi-weekly exercise.

We also observed increased RBCs and hemoglobin levels after dietary supplementation with MFGM. This result is consistent with our previous findings in mice [9]. The reason for the increased RBCs and hemoglobin after MFGM ingestion was unclear. MFGM is abundant in phospholipids that are critical components of the plasma membrane (Table 2). Decreased phospholipid content in the cell membrane of RBCs has been shown to attenuate the vulnerability of the cells [23], which may reduce cellular hemoglobin. One possible explanation is that absorbed phospholipids from MFGM might be incorporated into and stabilize the cell membranes of RBCs, hence retaining the hemoglobin. In fact, the number of RBCs tended to increase after MFGM ingestion for 4 weeks (Table 4). Because increased RBCs and hemoglobin improves oxygen transport and exercise performance [24], dietary supplementation with MFGM might also improve endurance capacity in humans. However, further studies are required to confirm the increase in RBCs and hemoglobin by dietary MFGM and clarify its underlying mechanism.

Several limitations of the present study should be considered. First, our sample size was small. Second, the washout period might be insufficient because the baseline value of the leg extension strength for period 2 was still higher than the baseline value for period 1. Third, the type and intensity of the exercise were also limited. Therefore, the effects of MFGM alone compared to those when combined with exercise of various types or intensities still need to be clarified. Finally, the dose-dependent nature of the beneficial effects of MFGM on muscles still requires investigation. Since activity of motor units varies with exercise intensity and type [25], we expect that dietary MFGM and exercise training would improve muscle function additively or synergistically. Studies comprising of larger cohorts are in progress to clarify these issues.

In conclusion, this study provides evidence that daily consumption of 1 g MFGM combined with regular exercise of moderate intensity improves muscle strength with increased motor unit activity, such as recruitment, in healthy Japanese middle-aged adult males. One gram of MFGM corresponds to 600 mL of milk; hence, absorbing adequate daily amounts of MFGM by consuming milk or dairy products may not be achievable. Therefore, daily supplementation with MFGM, together with regular exercise of moderate intensity, may be beneficial for the improvement of muscle function and physical performance.

## Abbreviations

ALT: Alanine aminotransferase
AST: Aspartate aminotransferase
Dok-7: Docking protein-7
EMG: Electromyography
MFGM: Milk fat globule membrane
MVC: Maximal voluntary contraction
NMJ: Neuromuscular junction
RBCs: Red blood cells
RMS: Root mean square
SEM: Standard error of the mean
